# Association of Various Spinopelvic Parameters and the Quality of Life in Those With Degenerative Lumbar Scoliosis in the Indian Population

**DOI:** 10.7759/cureus.40884

**Published:** 2023-06-24

**Authors:** Rameshwar Datt, Gunjar Jain, Anant Krishna, Vivek Vijayakumar, Shekhar Tank

**Affiliations:** 1 Orthopaedics, Employees' State Insurance (ESI) - Postgraduate Institute of Medical Sciences and Research (PGIMSR), New Delhi, IND; 2 Orthopaedics, All India Institute of Medical Sciences, Bhubaneswar, IND; 3 Orthopaedics, Maulana Azad Medical College, New Delhi, IND; 4 Orthopaedics, All India Institute of Medical Sciences, New Delhi, IND

**Keywords:** degenerative lumbar scoliosis, spinopelvic parameters, quality of life, indian population, coronal balance, sagittal balance

## Abstract

Purpose

The current study aimed to find any association between various spinopelvic parameters and the quality of life in patients with degenerative lumbar scoliosis (DLS) measured as per the Oswestry disability index (ODI) and visual analog scale (VAS) in the Indian population.

Methods

We included 20 DLS patients of more than 40 years with a Cobb angle of more than 10° and without any trauma, tumour, infection, or congenital scoliosis presented in our tertiary care centre in the study. The VAS and ODI were calculated for each patient. Radiological parameters were recorded for every patient, including sagittal balance, coronal balance, Cobb angle, pelvic incidence, pelvic tilt, and lumbar lordotic angle. We evaluated the results and sought any association between clinical and radiological variables for DLS.

Results

Patients with positive sagittal balance had significantly higher disability than those with neutral sagittal balance (p-value 0.007). Furthermore, patients with coronal imbalance had more severe pain (p-value 0.013) and disability (p-value 0.038) than those with neutral coronal balance. We also found that the ODI and VAS were not associated with any other spinopelvic parameters.

Conclusion

From the present study, we can conclude that in the Indian population, both positive sagittal and coronal imbalances are associated with poor functional status in patients with DLS. Therefore, while planning surgical correction for these patients, both coronal and sagittal balance are important and need to be considered.

## Introduction

Degenerative lumbar scoliosis (DLS) is a diverse disease complex affecting the spine in the elderly. It is characterized by more than 10 degrees of deformity in the coronal plane and a sagittal plane malalignment [1]. With a prevalence rate of 7.5% to 15%, it is one of the most common causes of structural scoliosis in patients older than 40 and a significant cause of backache, radiating pain, and disability in the geriatric population [1,2]. However, it is a common clinical finding that the severity of symptoms usually does not relate to the severity of the deformity. Also, not all patients with DLS have symptoms such as pain and disability. Therefore, the radiographic parameters associated with these patients' quality of life must be investigated.

Various studies to answer the above-mentioned query have given variable results. Some authors have shown a strong relationship between spinopelvic parameters and quality-of-life scores, whereas others have found only a weak correlation or none at all [3,4]. Some studies have found that only pelvic tilt affects spinal malalignment and clinical scores. In contrast, others have suggested sagittal balance as the sole criterion for consideration of clinical and functional scores in patients with DLS [5,6]. These studies were conducted for a Caucasian population; to our knowledge, no study has been undertaken for an Indian population. This dearth of knowledge and lack of consensus in the literature formed the basis for the present study. In this study, we aimed to ascertain if any radiological spinopelvic parameters are associated with the quality of life of patients with DLS in the Indian population.

## Materials and methods

The current study is a cross-sectional observational study conducted in the dedicated spine unit of our tertiary care center from January 2014 to March 2015. We obtained ethical clearance from the Institute Ethics Committee of Safdarjung Hospital and Vardhaman Mahavir Medical College (VMMC) before conducting the study (approval no. IEC/VMMC/SJH/Thesis/Nov-13/121). Twenty consecutive patients with DLS, aged more than 40 years, with a Cobb angle of more than 10 degrees, were included in the study. Written informed consent was obtained from each patient for their inclusion in the study. The diagnosis was made based on clinical examination, radiography, and magnetic resonance imaging. Patients with congenital or adolescent idiopathic scoliosis, spinal infection, spinal tumor, a history of previous spinal surgery or trauma, or any pathology that could influence the estimation of the patient's quality of life and severity of pain, e.g., hip osteoarthritis, were excluded from the study.

The authors conducted the clinical examination of patients in detail to rule out any other causes of scoliosis and any associated deformity in the pelvis or the limbs. It included an assessment of the back for swelling, deformity, scar, "café au lait" spots, sinus, fistula, or tuft of hair. The curve was named according to the side of convexity and the position of the major curve. We used the Oswestry disability index (ODI) and the visual analog scale (VAS) to evaluate the patient's quality of life and severity of back pain, respectively.

**Figure 1:**
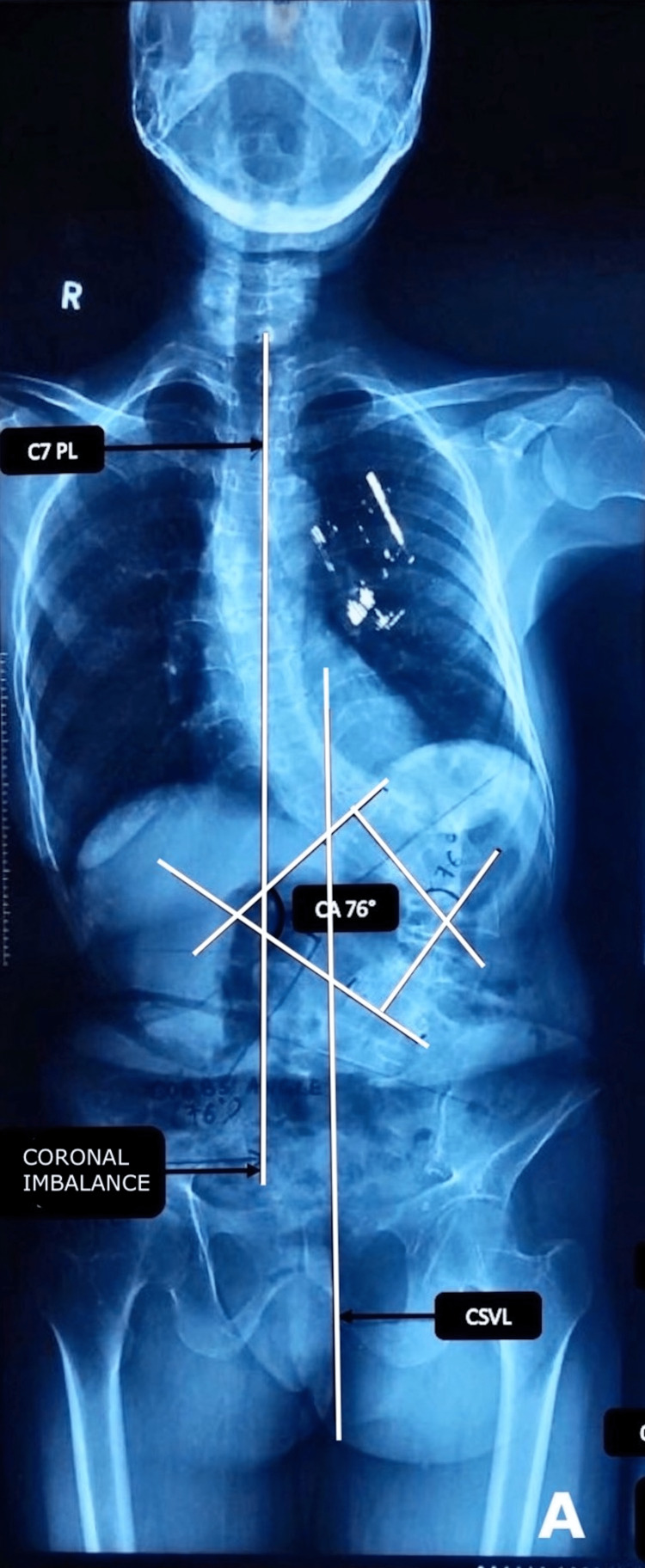
The 36-inch whole spine radiograph anteroposterior view shows a degenerative scoliotic deformity with a Cobb angle of 76° and a coronal imbalance C7PL: C7 plumb line, CA: Cobb angle, CSVL: Central sacral vertical line

**Figure 2:**
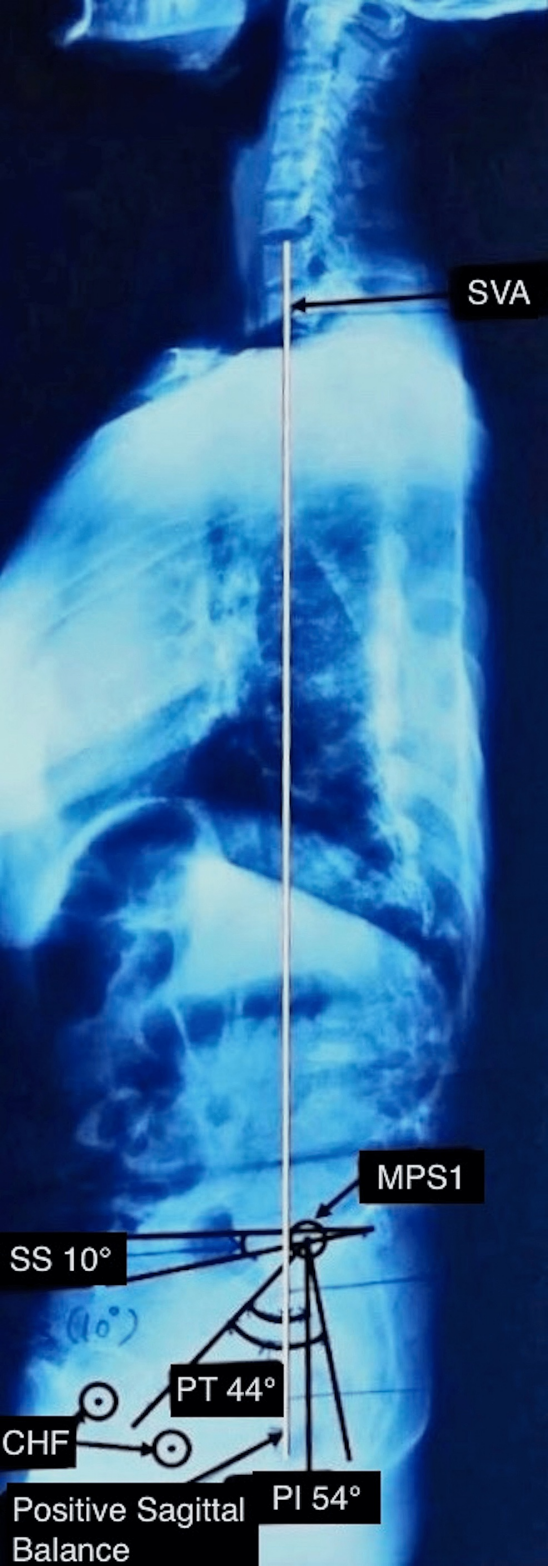
Whole spine lateral radiograph depicts a positive sagittal balance and the calculation of PT, SS, and PI PT: Pelvic tilt, SS: Sacral slope, PI: Pelvic incidence, SVA: Sagittal vertical axis, CFH: Centre of femoral heads, MPS1: Midpoint of the upper endplate of S1 vertebra

We obtained a 36-inch whole spine radiograph from the second cervical vertebra to the bilateral hip (sagittal profile) in the clavicle position of every patient, as described by Horton et al. [7], and a whole spine anteroposterior radiograph. The former was used for calculating sagittal balance, pelvic tilt, lumbar lordosis, pelvic incidence, and sacral slope (Figure 1). And the latter was used for calculating Cobb angle and coronal balance (Figure 2).

We documented sagittal balance as positive if the C7 plumbline passed anterior to the posterosuperior corner of the S1 vertebra and neutral when it passed through the posterosuperior corner of the S1 vertebra. The coronal imbalance was considered present if the C7 plumbline passed on either side of the central sacral vertical line.

Statistical analysis

The data obtained were analyzed using STATA 14.0 (StataCorp LLC, College Station, TX). Continuous variables, expressed in median ± standard deviation, were analyzed using a rank-sum or Kruskal-Wallis test. Moreover, categorical data, expressed as frequency and percentage, was assessed using the chi-square or Fisher's exact test. The correlation between the VAS and different continuous variables was analyzed by estimating the Pearson correlation coefficient. The criterion for statistical significance was a p-value less than 0.05.

## Results

Fifteen females and five males were included in the study. The mean age of all patients was 57 years (range 41 to 75). All the patients were under physical exercise therapy; 12 patients were using a thoracolumbar sacral brace; and three patients had undergone selective nerve root block six to eight months before the evaluation date, at either L4-L5 or L5-S1 levels.

Disability among the 20 patients, per the ODI questionnaire, was minimal in four, moderate in 10, and severe in six patients. Patients with positive sagittal balance had significantly higher disability than those with neutral sagittal balance (p-value 0.007, Table 1). Also, patients with coronal imbalance had more severe disabilities than those with neutral coronal balance (p-value 0.038, Table 2). Besides these two variables, we found no association between ODI and other radiological parameters, viz., sacral slope, Cobb angle, pelvic tilt, pelvic incidence, and lumbar lordosis (Table 3).

**Table 1:** The relation of ODI with sagittal balance ODI: Oswestry disability index, SB: Sagittal balance

SB	ODI			Total	P-value
	Mild	Moderate	Severe		
Positive	0 (0.0%)	5 (50%)	6 (100%)	11 (55%)	0.007
Neutral	4 (100.0%)	5 (50%)	0 (0.0 %)	9 (45 %)	
Total	4 (100.0%)	10 (100.0%)	6 (100.0%)	20 (100.0%)	

**Table 2:** The relation of the ODI with coronal balance ODI: Oswestry disability index, CB: Coronal balance

CB	ODI			Total	P-value
	Mild	Moderate	Severe		
Imbalance	1 (25%)	8 (80%)	6 (100%)	15 (75%)	0.038
Neutral	3 (75%)	2 (20%)	0 (0%)	5 (25%)	
Total	4 (100%)	10 (100%)	6 (100%)	20 (100.0%)	

**Table 3:** The relation between ODI and various parameters ODI: Oswestry disability Index, CI: Confidence interval, SE: Standard error

Variables	Minimum ODI (4 patients) Median ± SE	Moderate ODI (10 Patients) Median ± SE	Severe ODI (6 patients) Median ± SE	P-value
Age	47.5± 3.5	53.5 ± 3.8	72 ± 3.1	0.1973
Pelvic tilt (in degrees)	15.5± 3.5	24.5±3.8	31 ± 3.1	0.113
Pelvic incidence (in degrees)	50 ± 3.4	56.5 ± 3.8	54.5 ± 3.0	0.208
Sacral slope (in degrees)	32± 3.5	29.5 ± 3.8	28.5 ± 3.0	0.457
Lumbar lordosis (in degrees)	13 ± 3.5	25.5± 3.8	43 ± 3.0	0.184
Cobb angle (in degrees)	27.5 ± 3.5	21.0 ± 3.8	41.5 ± 3.0	0.522
Pelvic incidence-lumbar lordosis	37.5	30	13.5	0.178

The pain intensity, as measured through the VAS, was found to have a moderately positive correlation with pelvic tilt, lumbar lordosis, and Cobb angle and a moderately negative correlation with sacral slope (Table 4). Both positive sagittal balance and coronal imbalance were associated with a significantly higher VAS (Table 5).

**Table 4:** The relation between VAS and other variables VAS: Visual analog scale

Variables	Pearson correlation coefficient (r)	P-value
Age	0.1747	0.4614
Pelvic tilt	0.3654	0.1132
Pelvic incidence	0.052	0.824
Sacral slope	-0.3504	0.129
Lumbar lordosis	0.431	0.057
Cobb angle	0.3206	0.1682
Pelvic incidence-lumbar lordosis	-0.3866	0.092

**Table 5:** Relation of VAS with sagittal and coronal balance VAS: Visual analog scale

	Median VAS (SD)	P-value
Patients with Positive sagittal balance	6 (1.3)	0.002
Patients with Neutral sagittal balance	4 (0.5)	
Patients with Neutral coronal balance	4 (0.44)	0.013
Patients with Coronal imbalance	5 (1.35)	

## Discussion

In our study, all patients with positive sagittal balance had moderate to severe disability, and the association between these variables was statistically significant (p-value 0.007). Most previous studies, including the ones by Thiong et al. and Glassman et al., have reported a similar association between these two variables [8,9]. However, Ploumis et al. reported no correlation between positive sagittal balance and the ODI, primarily because they considered sagittal balance to be positive when the C7 plumb line lies more than 5 cm anterior to the posterosuperior corner of the S1 vertebra [10]. However, most of the investigations, including the one by the current authors, have defined the sagittal balance as positive when the C7 plumbline lies anywhere anterior to the posterosuperior corner of the S1 vertebra [8,9].

Similar to our finding, Jeon et al., in their study, also found a significant association between ODI and coronal imbalance [11]. Our study suggests that coronal and sagittal imbalances are associated with poor functional status. However, most studies suggest that spinal deformity in the sagittal plane is more critical than coronal plane deformity [9]. The authors believe this finding could be due to the cultural differences in the daily activities of the Indian population compared to that of the Caucasian population, viz., sitting cross-legged, deep squatting, frequently working in a stooped posture, and carrying a load on the head. Thus, demographical and geographical factors may affect the functional status of patients with DLS, and further research can be conducted.

The authors of the present study observed that the severity of pelvic tilt had a moderately positive correlation with disability, though the findings were not statistically significant. This finding is in concordance with studies by Schwab et al. and Lafage et al. and can be explained by the fact that the patient compensates for the spinal deformity in the sagittal plane by increasing the pelvic tilt [6,12,13]. So, increased pelvic tilt is a proxy indicator of malalignment in the sagittal plane.

Schwab et al. and Boissiere et al. found that the mismatch between pelvic incidence and lumbar lordosis contributes to disability [6,14]. In our study, though there was a moderately negative correlation between disability and pelvic incidence-lumbar lordosis and a moderately positive correlation between lumbar lordosis and ODI, both were not statistically significant. Results similar to ours have been documented by Ha et al. [15]. Therefore, the current authors believe these parameters play a minor role in determining the disability of patients with DLS.

The Cobb angle measures the degree of deformity in the coronal plane, and a higher angle indicates a severer deformity. However, the current study's authors found no association between the Cobb angle and the degree of disability. Variable results are found in the literature, with some reporting no correlation and others reporting a positive correlation between Cobb angle, pain intensity, and degree of disability. Our finding is supported by the work of Ha et al. [15].

In our study, no other spinopelvic parameters were associated with increased pain except for coronal imbalance and positive sagittal balance. Variable findings have been reported in the literature in this regard. Gao et al. and Schwab et al. found that only lumbar lordosis was significantly associated with pain [3,16]. However, in the study by Lizuka et al., the sacral slope had a bearing on VAS [4].

It should be noted that the present study has a few limitations. The primary limitation is the study design, i.e., a single-center cross-sectional observational study. Therefore, a temporal relationship between the parameters cannot be evaluated. Also, the behavior of the parameters over time remains suspicious. The major limitation of our study was the limited sample size. We have selected a low sample size based on the patient load in our outpatient department to complete the study in the stipulated time. Though the sample size is too small to extrapolate our findings to the general population, our study holds significance as it is the first study of the Indian population and will guide future studies. Since our study is a single center with a small sample size, the statistical power of the study remains low, and the external validity is also limited. Furthermore, patients with DLS who remained asymptomatic and did not report to the outpatient clinic could not be included in our study. A selective nerve root block was performed on three patients, which the authors believe might have influenced the results in these patients to some extent.

## Conclusions

From the present study, we can conclude that in the Indian population, both positive sagittal balance and coronal imbalance are associated with poor functional status in patients with DLS. Therefore, the coronal and sagittal balance must be considered while planning the surgical correction of these patients. Based on our results, future multicenter studies with larger sample sizes need to be performed to validate our results further.
